# Risk factors for perioperative hidden blood loss after one-segment posterior circumferential decompression surgery on thoracic ossification of the posterior longitudinal ligament: a finding of the double-layer sign on CT

**DOI:** 10.1186/s12891-023-06352-7

**Published:** 2023-03-24

**Authors:** Huiqiang Liang, Xuan Zhao, Linfeng Wang, Jia Li, Yong Shen

**Affiliations:** 1 Department of Orthopedic Surgery, Third Hospital of Hebei Medical University, Shijiazhuang, 050051 People’s Republic of China; 2 The Key Laboratory of Orthopedic Biomechanics of Hebei Province, The Third Hospital of Hebei Medical University, Shijiazhuang, 050051 People’s Republic of China

**Keywords:** Hidden blood loss (HBL), Risk factors, Thoracic ossification of the posterior longitudinal ligament (T-OPLL), Double-layer sign, Posterior circumferential decompression, Multivariate linear regression analysis

## Abstract

**Background:**

Hidden blood loss (HBL) is of increasing interest to spine surgeons. This retrospective study aimed to evaluate perioperative HBL and its risk factors in patients undergoing one-segment posterior circumferential decompression surgery on thoracic ossification of the posterior longitudinal ligament (T-OPLL).

**Method:**

We retrospectively studied 112 patients diagnosed with T-OPLL following posterior circumferential decompression surgery from August 2015 to June 2020. Patient demographics, blood loss-related parameters, surgery-related data and imaging parameters were extracted. Postoperative complications were also recorded. Pearson or Spearman correlation analysis was used to investigate the correlation between patient demographics and HBL. Multivariate linear regression analysis was performed to determine the independent risk factors associated with HBL.

**Results:**

Forty-five men and 67 women were involved in this research, with an average age of 56.4 ± 10.2 years. The mean HBL was 459.6 ± 275.4 ml, accounting for 56.5% of the total blood loss. Multiple linear regression analysis showed that double-layer sign (*P* = 0.000), ossification occupancy ratio (OOR) > 60% (*P* = 0.030), age (*P* = 0.010), hematocrit (Hct) loss (*P* = 0.034), and postoperative Hct (*P* = 0.016) were independent risk factors for HBL. However, OPLL morphology (*P* = 0.319), operation time (*P* = 0.587), hemoglobin (Hb) loss (*P* = 0.644), and postoperative Hb (*P* = 0.952) were not significantly different from HBL.

**Conclusion:**

A high proportion of HBL was found after posterior circumferential decompression surgery on T-OPLL during the perioperative period, which should not be overlooked. Double-layer sign, OOR > 60%, age, Hct loss and postoperative Hct are independent risk factors for HBL.

## Introduction

Spinal fusion surgery can lead to severe injury to soft tissues and muscles, accompanied by significant blood loss, which requires proper management [1, 2]. In fact, patients usually have a degree of anemia after surgery that is inconsistent with the amount of blood loss during surgery, which is due to hidden blood loss (HBL). Some research has reported that HBL can be associated with increased blood loss and other complications that may contribute to poor outcomes [3, 4]. HBL is usually ignored by spine surgeons because of its invisibility and therefore difficult detection. In 2000, the concept of HBL was first put forward by Sehat et al. [5], who reported that HBL accounted for 49% of total blood loss after total hip arthroplasty. Gradually, some scholars successively reported results about HBL in spine surgery, such as adolescent idiopathic scoliosis surgery [6], oblique lateral interbody fusion [7], thoracolumbar fracture [8], minimally invasive transforaminal interbody fusion [9] and percutaneous endoscopic transforaminal discectomy [10]. The concept of HBL has attracted increasing attention from spine surgeons in recent years.

Thoracic ossification of the posterior longitudinal ligament (T-OPLL) is a progressive disease characterized by heterotopic ossification of the ligaments. The accompanying spinal cord compression often leads to severe neurological dysfunction. Once symptoms appear, conservative treatment is ineffective, and surgery is the only treatment. The incidence of T-OPLL was approximately 1.9% [11]. Due to anatomical factors of the thoracic spine, surgical treatment of T-OPLL is a great challenge in the field of spinal surgery. To date, various surgical techniques have been developed to treat T-OPLL, especially posterior circumferential decompression, which has gradually become accepted by spine surgeons because of the good recovery of nerve function [12–14]. Such a surgical procedure can directly remove the pressure substance in front of the spinal cord to achieve direct decompression. However, research has indicated that the incidence of complications in posterior circumferential decompression is much higher than that in single posterior decompression with more blood loss, although there are no significant differences in neurological recovery [15].

To the best of our knowledge, no research has specifically analyzed the risk factors for HBL in patients diagnosed with T-OPLL following posterior circumferential decompression surgery. We retrospectively collected the clinical data of patients diagnosed with T-OPLL who underwent one-segment posterior circumferential decompression surgery in our hospital and evaluated HBL and its risk factors.

## Materials and methods

### Ethics statement

All experimental protocols were approved by the ethics committee of The Third Hospital of Hebei Medical University and all methods were performed in accordance with the Declaration of Helsinki. Ethics committee of the The Third Hospital of Hebei Medical University waived the need for informed consent as data was anonymized before the analyses.

### Study design and patient population

From August 2015 to June 2020, 251 patients diagnosed with T-OPLL underwent thoracic decompression surgery at the Third Hospital of Hebei Medical University. The inclusion criteria were as follows: (1) patients who met the clinical diagnostic criteria for T-OPLL and suffered from chronic thoracic spinal cord compression, mainly presenting as follows: vague chest and back pain, hypoesthesia of the lower limbs and body, presence of hypoesthesia plane, decreased muscle strength of both lower limbs, hyperreflexia of tendons of both lower limbs, instability of walking (with a feeling of stepping on the cotton), and urinary and bowel dysfunction; (2) patients whose CT suggested thoracic ossification of the posterior longitudinal ligament and MRI indicated spinal cord compression; (3) patients who underwent posterior circumferential decompression surgery; and (4) those with a one-segment surgical level. The exclusion criteria were as follows: (1) patients with coagulation disorders; (2) patients who were treated with anticoagulant drugs; (3) patients who experienced intraoperative blood loss greater than 2.5 L causing larger bias [16]; (4) patients who suffered cerebrospinal fluid leakage; (5) those who have previously undergone a thoracic surgery; and (6) those with thoracic infections or tumors. On the basis of the aforementioned criteria, 112 patients (52 males and 60 females) were enrolled in this study. With respect to the performed level, 25 patients underwent surgery at the T4-T5 level, 33 at the T5-T6 level, 26 at the T6-T7 level, and 28 at the T7-T8 level.

### Surgical technique

All posterior circumferential decompression surgeries were performed by the same experienced spine surgeon (Y.S.). Continuous intraoperative neurophysiological monitoring was applied. The mean arterial blood pressure > 90 mmHg was maintained during surgery to optimize spinal cord perfusion.

The patients were placed in the prone position with their backs fully exposed, and oral tracheal intubation was performed under general anesthesia. After confirming the decompression segment and surgical exposure range of the C-arm before the operation, a midline incision was made on the skin of the posterior chest and back. The thoracic paravertebral muscles were dissociated to expose the lamina, lateral edge of the articular process and base of the transverse process. Pedicle screws were placed into the vertebral body one level above and below the circumferential decompression segment. A high-speed drill and ultrasonic bone scalpel were used to carefully notch the junction of the bilateral lamina and articular process to the medial cortical layer. Nerve stripping ions were used to separate the bilateral lamina and the inferior ligamentum flavum, and the decompressed vertebral lamina was uncovered.

After the posterior wall of the spinal canal was uncovered and removed and dorsal decompression was completed, both articular processes were removed using the ultrasonic bone scalpel, and the pedicle was cut to the level of the posterior edge of the vertebral body. Then, an ultrasonic bone scalpel and a curet were used for notching to eliminate the cancellous bone between the two grooves. After a "culvert" was created in the vertebral body, an "L" osteotomy knife was placed into the "safe triangle" on both sides of the ossified posterior longitudinal ligament. After fully protecting the nerve roots, we used bipolar electrocoagulation for intraoperative bleeding to achieve hemostasis, and cotton slivers were applied to stop bleeding from the venous plexus in front of the dura mater. The adhesion between the dura mater and pressor was severed by a sharp knife. The ossified posterior longitudinal ligament was cut and removed to achieve anterior decompression of the spinal cord. The articular process joints were thinned to the cancellous bone using a grinding drill, and posterolateral bone grafting and fusion were performed.

### Clinical and radiographic measures

Demographic features and operative data, such as age, sex, body mass index (BMI), comorbidity (hypertension, diabetes), operation time and performed level were recorded. Blood loss-related parameters included the patient’s blood volume, intraoperative blood loss, preoperative hemoglobin (Hb), preoperative hematocrit (Hct), postoperative Hb, and postoperative Hct. Hemoglobin concentration was used to determine the presence of anemia (i.e., < 120 g/l for women and < 130 g/l for men) [17].

**Fig. 1:**
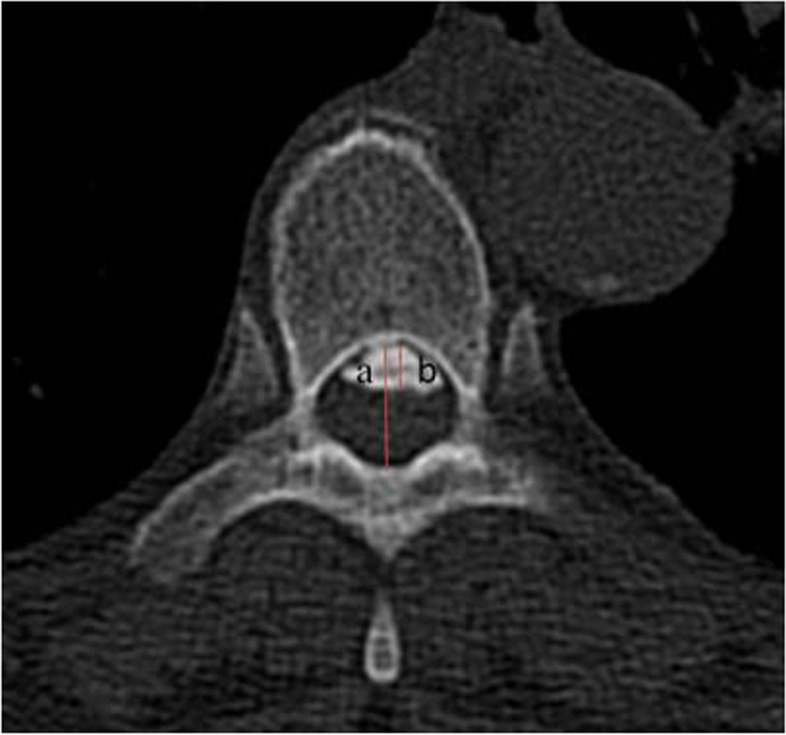
OOR: the OPLL diameter of the thickest ossified part (**b**) divided by the spinal canal diameter (**a**) × 100%

**Fig. 2:**
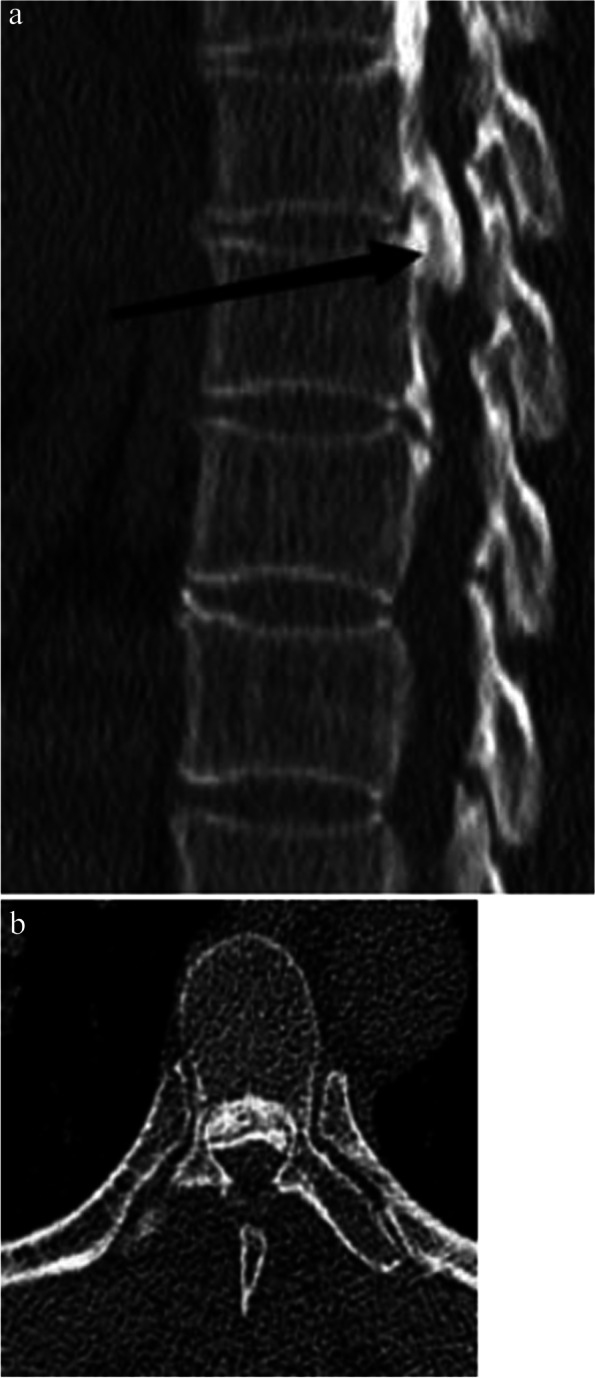
**a** sagittal bone window CT demonstrating the double-layer sign in T-OPLL. **b** axial bone window CT demonstrating the double-layer sign in T-OPLL

Radiographic measures included thoracic kyphosis, OPLL morphology, OOR and double-layer sign. The evaluation was performed by two professional radiologists who did not know the clinical results. The preoperative thoracic kyphosis angle was defined as the Cobb angle between the T4 upper endplate and the T12 lower endplate. According to the OPLL morphology on the sagittal imaging of CT before the operation, T-OPLL was divided into flat-type and beak-type. The OOR was defined as the OPLL diameter of the thickest ossified part divided by the spinal canal diameter **(**Fig. 1). According to the imaging research of OPLL [18, 19], the double-layer sign is observed on bone window CT, which is characterized by high-density ossification with the front and rear edges separated by a central low-density mass, indicating that it is significantly related to dural ossification (Fig. 2). MRI was used to predict the degree of spinal cord compression.

### Blood loss management and calculation of HBL

Intraoperative blood loss was considered as the blood in the inhaled container plus the blood soaked with sponge. The postoperative drainage volume was calculated by measuring the blood volume in the drainage bottle before removing the drainage tube on the second or third day after the operation. The study confirmed that the hemodynamics of patients at this time were stable [20]. When calculating the blood loss, if the drainage tube could not be removed, we measured the blood volume of the drainage tube on the day of blood extraction. Intraoperative lost blood could be collected by use of cell saver and reinfused into the patient as autologous blood after filtration on the basis of the patient's condition during operation. Patients with intraoperative hemoglobin lower than 90 g/L or hypotension were given a blood transfusion. We followed the method used by Nadler et al. [21] to estimate preoperative blood volume (PBV) based on sex, height (m) and weight (kg), PBV = [k1 × height^3^] + [k^2^ × weight] + k3, in which the k1, k2 and k3 of males (females) were 0.3669 (0.3561), 0.03219 (0.03308) and 0.6041 (0.1833), respectively. The method described by Gross et al. [22] was used to estimate the total blood loss (TBL), with TBL = PBV × (Hct_preop_ − Hct_postop_)/average (Hct_preop_ + Hct_postop_), where Hct_preop_ is Hct before the operation, Hct_post_ is Hct on the second or third day after the operation, and Hct_ave_ is the average value of Hct_preop_ and Hct_post_. Measured blood loss (MBL) = blood volume in the inhaled container + blood volume of soaked sponge + postoperative drainage volume. The method of Sehat et al. [20] was used to calculate the hidden blood loss (HBL), where HBL = total blood loss—measured blood loss. When transfusion was performed, the method was modified as follows: HBL = TBL + blood infusion volume − MBL.

### Postoperative Complications

According to similar studies [23], we counted postoperative complications within the seventh postoperative day, such as wound infection, wound damage, acute heart failure, spinal epidural hematoma, deep venous thrombosis, pneumonia, pulmonary embolism and > 4 units of red blood cell transfusion within 72 h.

### Statistical analysis

IBM SPSS 26.0 software (IBM Corp., Armonk, NY, USA) was used for statistical analysis. The chi-squared test was used to compare preoperative and postoperative anemia. Student's t test was used to compare the difference between Hb levels and Hct values before and after the operation. Pearson correlation analysis (for normal data), Spearman correlation analysis (for nonnormal data) and multiple linear regression analysis were used to explore the risk factors related to HBL. The level of statistical significance was set at *P* < 0.05.

## Results

The data of 112 patients (52 males and 60 females) were retrospectively analyzed. Regarding the surgical level, 25 patients underwent surgery at the T4-T5 level, 33 at the T5-T6 level, 26 at the T6-T7 level, and 28 at the T7-T8 level. The demographic characteristics of the patients are summarized in Table 1. The mean age was 56.4 ± 10.2 years, and the mean BMI was 24.5 ± 3.2 kg/m2. The OPLL morphology of 78 patients was flat-type, whereas 34 patients were beak-type. A total of 31 patients had OOR > 60% compared with 81 patients who did not. The mean thoracic kyphosis angle was 11.8 ± 4.5°, and the mean operation time was 245.6 ± 49.3 min. Thirteen patients showed the double-layer sign on CT. The mean MBL was 353.3 ± 105.2 ml, the mean HBL was 459.6 ± 275.4 ml, the mean TBL was 812.8 ± 299.8 ml, the mean preoperative Hct and Hb were 37.8 ± 4.2% and 122.1 ± 15.5 g/l, respectively, the mean postoperative Hct and Hb were 31.7 ± 4.3% and 106.9 ± 15.7 g/l, respectively, Hct loss was 6.6 ± 2.4%, and Hb loss was 15.8 ± 7.2 g/l. A total of 34 patients with normal hemoglobin before the operation showed anemia after the operation (*p* < 0.001, Table 2).

**Table 1:** Patient demographics

Parameters	Statistics
Total patients (*n*)	112
Sex (M/F)	52/60
Age, yr	56.4 ± 10.2
BMI, kg/m2	24.5 ± 3.2
Hypertension (n)	29
Diabetes mellitus (n)	24
OPLL morphology(n)	
Flat-type	78
Beak-type	34
MBL, ml	353.3 ± 105.2
HBL, ml	459.6 ± 275.4
TBL, ml	812.8 ± 299.8
HBL/TBL (%)	56.5 ± 12.7
Hct loss(%)	6.6 ± 2.4
Hb loss(%)	15.8 ± 7.2
Preoperative Hct	37.8 ± 4.2
Preoperative Hb, g/l	122.1 ± 15.5
Postoperative Hct	31.8 ± 4.3
Postoperative Hb, g/l	106.9 ± 15.7
Thoracic kyphosis, °	11.8 ± 4.5
OOR, %	48.3 ± 14.2
≤ 60	81
> 60	31
Operation time, min	245.6 ± 49.3
Double-layer sign	
Yes	13
No	99
Postoperative complications	12
Performed level	
T4-T5	25
T5-T6	33
T6-T7	26
T6-T8	28

*HBL* Hidden blood loss, *TBL* Total blood loss, *MBL* Measured blood loss, *Hct* Hematocrit, *Hb* Hemoglobin, *BMI* Body mass index, *OOR* Ossification occupancy ratio

**Table 2:** Changes in Hct, Hb and anemia level following posterior circumferential decompression surgery

	Preoperative (*n* = 112)	Postoperative (*n* = 112)	Statistical significance
Hct, %	37.8 ± 4.2	31.7 ± 4.3	*P* < 0.001
Hb, g/l	122.1 ± 15.5	106.9 ± 15.7	*P* < 0.001
Anemia	62	96	*P* < 0.001

*Hct* Hematocrit, *Hb* Hemoglobin

Through Pearson or Spearman correlation analysis for HBL, the following parameters were statistically significant (Table 3): age (*P* = 0.000), OPLL morphology (*P* = 0.034), Hct loss (*P* = 0.000), Hb loss (*P* = 0.015), postoperative Hct (*P* = 0.000), postoperative Hb (*P* = 0.000), OOR (*P* = 0.000), operation time (*P* = 0.026), postoperative complications (*P* = 0.000), and double-layer sign (*P* = 0.000). To determine the relationship between HBL and possible risk factors, multiple linear regression analysis was performed. It confirmed that, among all variables, age (*P* = 0.010), Hct loss (*P* = 0.034), postoperative Hct (*P* = 0.016), OOR > 60% (*P* = 0.030), and double-layer sign (*P* = 0.000) were statistically significant as independent risk factors for HBL (Table 4).

**Table 3:** Results of the Pearson or Spearman correlation analysis for HBL

Parameters	Sig (2-tailed)	P
Sex	0.048	0.632
Age	0.785	**0.000**
BMI	0.095	0.347
Hypertension	0.076	0.510
Diabetes mellitus	0.082	0.411
OPLL morphology	0.175	**0.034**
Hct loss	0.779	**0.000**
Hb loss	0.191	**0.015**
Postoperative Hct	-0.468	**0.000**
Postoperative Hb	-0.395	**0.000**
MBL	0.146	0.082
Thoracic kyphosis	0.086	0.414
OOR	0.418	**0.000**
Operation time	0.180	**0.026**
Double-layer sign	0.962	**0.000**
Postoperative complications	0.733	**0.000**
Performed level		
T4-T5	0.096	0.135
T5-T6	0.114	0.108
T6-T7	0.058	0.662
T7-T8	0.072	0.354

Values in bold indicate statistical significance

*MBL* Measured blood loss, *Hct* Hematocrit, *Hb* Hemoglobin, *BMI* Body mass index, *OOR* Ossification occupancy ratio

**Table 4:** Results of multivariate linear regression for HBL

Coefficients*	Unstandardizedβ	SE	Unstandardizedβ	t	*P*
Constant	82.432	105.451		0.782	0.536
Age	7.436	2.522	0.198	2.948	**0.010**
OPLL morphology	-5.855	4.797	-0.064	-1.221	0.319
Hct loss	4.257	2.090	0.056	-2.137	**0.034**
Hb loss	0.788	1.093	0.039	0.721	0.644
Postoperative Hct	-24.684	9.114	-0.322	2.708	**0.016**
Postoperative Hb	0.132	0.308	0.006	0.429	0.952
OOR > 60%	62.151	28.085	0.147	2.213	**0.030**
Postoperative complications	-20.663	15.424	-0.078	-1.340	0.092
Operation time	-0.616	0.812	-0.124	0.759	0.587
Double-layer sign	5.429	1.428	0.155	3.802	**0.000**

Values in bold indicate statistical significance

^*^Dependent variable: hidden blood loss (ml)

*HBL * Hidden blood loss, *Hct * Hematocrit, *Hb * Hemoglobin, *OOR *Ossification occupancy ratio

No neurologic impairment occurred in all patients involved after operation. Postoperative complications within 7 days occurred in 12 patients (11% of the total patients), including 3 cases of wound infection, 2 cases of wound damage, 1 case of deep venous thrombosis, 2 cases of spinal epidural hematoma, 1 case of acute heart failure, 1 case of pneumonia and 2 cases of > 4 units of red blood cell transfusion within 72 h. After timely detection and treatment, there was no death or reoperation, the patients' postoperative complications were effectively treated, and they were safely discharged. Multiple linear regression analysis suggested that postoperative complications were not significantly associated with HBL.

## Discussion

Excessive blood loss often occurs during spine fusion surgery [24], but the reasons for the occurrence of HBL are unclear. A study using labeled red blood cells showed that the main source of HBL is the extravasation of blood into tissues [25]. In 2000, Sehat et al. [5] put forward the concept of HBL for the first time. Later, an increasing number of spinal surgeons began recognizing HBL. In recent years, Jiang C et al. [4] believed that the mean HBL of patients receiving cervical open-door laminoplasty was approximately 337.2 ± 187.8 ml, which was 46.8% of TBL. Wang H et al. [26] held that the actual amount of HBL in patients undergoing unilateral biportal endoscopic spine surgery was 469.5 ± 195.3 ml, accounting for 57.6% of TBL. The research conducted by Ge M et al. [27] suggested that the amount of HBL is even higher than previously appreciated (endoscopic transforaminal lumbar interbody fusion: 717.9 ± 220.1 ml; minimally invasive transforaminal lumbar interbody: 942.3 ± 219.1 ml). Our research also showed a substantial amount of HBL, which was 459.6 ± 275.4 ml, representing 56.5% of TBL. To date, the influencing factors of posterior circumferential decompression surgery on T-OPLL related to HBL have not been confirmed. In this study, clinical information from 112 patients with T-OPLL who underwent circumferential decompression surgery was retrospectively analyzed to evaluate and identify risk factors for HBL by multiple linear regression.

Multiple linear regression analysis showed that double-layer sign on bone window CT was an important factor for HBL of posterior circumferential decompression surgery as a treatment for T-OPLL. The double-layer sign, characterized by anterior and posterior ossified masses separated by a central hypodense mass (hypertrophied but nonossified posterior longitudinal ligament), appeared more specific for dural ossification [28]. There were prominent thick-walled vessels, predominantly venules, around the nonossified posterior longitudinal ligament accompanied by dilated perivascular spaces [19]. It is recognized that during posterior circumferential decompression, additional ventral decompression of the spinal cord implies removal of the pressure substance in front, which can provide sufficient space for the spinal cord [11, 12]. We thought that the venous plexus around the nonossified posterior longitudinal ligament was inevitably destroyed during this process, and blood flowed to and remained in the soft tissue space, resulting in an increase in HBL. Meanwhile, through multiple linear regression analysis, OPLL morphology was not found to be related to HBL.

In this study, we investigated OOR > 60% was an independent risk factor for HBL. High epidural pressure is an important risk factor for intraoperative blood loss [29]. Due to the existence of an ossified mass, the local venous reflux is not sufficient, high venous pressure leads to thin wall of venous plexus, and the increase in epidural pressure will further lead to an increase in vascular tension in the surrounding tissues, ultimately increasing bleeding of the surrounding tissues during the operation. Some of this oozing blood is visible to the naked eye, while the other part may enter the surrounding tissue space and become an important part of HBL.

Our study showed a significant positive correlation between age and HBL. Previous research findings have reported that age is a risk factor for HBL during posterior lumbar fusion [26, 30]. Our final result was consistent with that observation. On the one hand, elderly patients may have poor vascular regulation ability and are prone to vascular sclerosis, resulting in high vascular brittleness, easily damaged vascular walls and increased capillary permeability. On the other hand, due to malnutrition, accelerated muscle loss and soft tissue relaxation in elderly patients, bleeding may be more likely to seep into the perivascular stroma.

Hct loss and postoperative Hct were considered independent factors for HBL, and postoperative Hct was negatively correlated with HBL. The locations of Hct loss were divided into MBL and HBL. However, we did not find a correlation between MBL and HBL. Through Student’s t test, we found significant differences between Hct and Hb before and after the operation. More red blood cells were lost during continuous blood loss, and previous research showed that Hct was reduced in combination with infusion dilution [31], which might have contributed to more Hct changes. This may explain why Hb and Hct showed different results in multiple linear regression analysis.

Perioperative reduction of blood loss, especially HBL, has become the focus of orthopaedic surgeons. We believe that this study has certain reference value for subsequent related research about posterior circumferential decompression surgery on thoracic ossification of the posterior longitudinal ligament.

Several limitations should be noted in our study. First, this was a single-center retrospective study, and the number of patients was relatively small. Second, liquid equilibrium is an important component when calculating HBL. However, because of the lack of specific rehydration parameters, the conclusions were limited. Finally, because most patients enrolled in our research were native residents, the influence of racial and regional differences on HBL was not investigated. Therefore, high-quality observational studies and further validation are needed.

## Conclusions

In conclusion, there is a substantial portion of HBL in patients diagnosed with T-OPLL following posterior circumferential decompression surgery. Double-layer sign, OOR > 60%, age, Hct loss and postoperative Hct were independent risk factors. During the perioperative period, more attention should be given to HBL, and its risk factors should be evaluated according to a patient's particular situation. Spine surgeons should be cognizant of HBL and enhance appropriate perioperative fluid management.

## Data Availability

The datasets generated and analysed during the current study are not publicly available due to hospital policies but are available from the corresponding author on reasonable request.

## References

[1] Quarto E, Bourret S, Rebollar Y, Mannem A, Cloche T, Balabaud L, Boue L, Thompson W, Le Huec JC (2022). Team management in complex posterior spinal surgery allows blood loss limitation. Int Orthop.

[2] Le Huec JC, AlEissa S, Bowey AJ, Debono B, El-Shawarbi A, Fernández-Baillo N, Han KS, Martin-Benlloch A, Pflugmacher R, Sabatier P, Vanni D, Walker I, Warren T, Litrico S (2022). Hemostats in Spine Surgery: Literature Review and Expert Panel Recommendations. Neurospine.

[3] Ogura Y, Dimar Ii JR, Gum JL, Crawford CH, Djurasovic M, Glassman SD, Carreon LY (2019). Hidden blood loss following 2- to 3-level posterior lumbar fusion. Spine J.

[4] Jiang C, Chen TH, Chen ZX, Sun ZM, Zhang H, Wu YS (2019). Hidden blood loss and its possible risk factors in cervical open-door laminoplasty. J Int Med Res.

[5] Sehat KR, Evans R, Newman JH (2000). How much blood is really lost in total knee arthroplasty?. Correct blood loss management should take hidden loss into account. Knee.

[6] Wang L, Liu J, Song X, Luo M, Chen Y (2021). Hidden blood loss in adolescent idiopathic scoliosis patients undergoing posterior spinal fusion surgery: a retrospective study of 765 cases at a single centre. BMC Musculoskelet Disord.

[7] Zhu L, Zhang L, Shan Y, Feng X, Zhang W (2021). Analysis of Hidden Blood Loss and its Risk Factors in Oblique Lateral Interbody Fusion Surgery. Clin Spine Surg.

[8] Yue X, Zhang J, Sun T, Zhang W, Yang M, Li Z (2022). Hidden blood loss and its influencing factors after minimally invasive percutaneous transpedicular screw fixation in thoracolumbar fracture. BMC Musculoskelet Disord.

[9] Zhou Y, Fu X, Yang M, Ke S, Wang B, Li Z (2020). Hidden blood loss and its possible risk factors in minimally invasive transforaminal lumbar interbody fusion. J Orthop Surg Res.

[10] Hu M, Zhang Y, Zhao WJ, Liu X, Shi PZ, Wang JW, Cai TC, Zhang L (2022). Perioperative Hidden Blood Loss in Lumbar Disk Herniation Patients With Percutaneous Endoscopic Transforaminal Discectomy and Influencing Factors. Clin Spine Surg.

[11] Mori K, Imai S, Kasahara T, Nishizawa K, Mimura T, Matsusue Y (2014). Prevalence, distribution, and morphology of thoracic ossification of the posterior longitudinal ligament in Japanese: results of CT-based cross-sectional study. Spine (Phila Pa 1976).

[12] Kanno H, Aizawa T, Hashimoto K, Itoi E, Ozawa H (2021). Anterior decompression through a posterior approach for thoracic myelopathy caused by ossification of the posterior longitudinal ligament: a novel concept in anterior decompression and technical notes with the preliminary outcomes. J Neurosurg Spine.

[13] Kato S, Murakami H, Demura S, Yoshioka K, Yokogawa N, Takaki S, Oku N, Tsuchiya H (2020). Indication for anterior spinal cord decompression via a posterolateral approach for the treatment of ossification of the posterior longitudinal ligament in the thoracic spine: a prospective cohort study. Eur Spine J.

[14] Chen G, Chen Z, Li W, Zeng Y, Zhong W, Sun C (2020). "IV+V+VI" Circumferential Decompression Technique for Thoracic Myelopathy Caused by the Ossification of Posterior Longitudinal Ligament or Hard Disc Herniation.. Spine (Phila Pa 1976).

[15] Zhu S, Wang Y, Yin P, Su Q (2020). A systematic review of surgical procedures on thoracic myelopathy. J Orthop Surg Res.

[16] Smorgick Y, Baker KC, Bachison CC, Herkowitz HN, Montgomery DM, Fischgrund JS (2013). Hidden blood loss during posterior spine fusion surgery. Spine J.

[17] Smilowitz NR, Oberweis BS, Nukala S, Rosenberg A, Zhao S, Xu J, Stuchin S, Iorio R, Errico T, Radford MJ, Berger JS (2016). Association Between Anemia, Bleeding, and Transfusion with Long-term Mortality Following Noncardiac Surgery. Am J Med.

[18] Min JH, Jang JS, Lee SH (2007). Significance of the double- and single-layer signs in the ossification of the posterior longitudinal ligament of the thoracic spine. Neurosurgery.

[19] Mizuno J (2018). Radiologic Evaluation of Ossification of the Posterior Longitudinal Ligament with Dural Ossification. Neurosurg Clin N Am.

[20] Sehat KR, Evans RL, Newman JH (2004). Hidden blood loss following hip and knee arthroplasty. Correct management of blood loss should take hidden loss into account. J Bone Joint Surg Br.

[21] Nadler SB, Hidalgo JH, Bloch T (1962). Prediction of blood volume in normal human adults. Surgery.

[22] Gross JB (1983). Estimating allowable blood loss: corrected for dilution. Anesthesiology.

[23] Zhang R, Xing F, Yang Z, Lin G, Chu J (2020). Analysis of risk factors for perioperative hidden blood loss in patients undergoing transforaminal lumbar interbody fusion. J Int Med Res.

[24] Kim HB, Kweon TD, Chang CH, Kim JY, Kim KS, Kim JY (2021). Equal Ratio Ventilation Reduces Blood Loss During Posterior Lumbar Interbody Fusion Surgery. Spine (Phila Pa 1976).

[25] Ren Z, Li S, Sheng L, Zhuang Q, Li Z,  Xu D, Chen X, Jiang P, Zhang X (2017). Efficacy and Safety of Topical Use of Tranexamic Acid in Reducing Blood Loss During Primary Lumbar Spinal Surgery: A Retrospective Case Control Study. Spine (Phila Pa 1976).

[26] Wang H, Wang K, Lv B, Li W, Fan T, Zhao J, Kang M, Dong R, Qu Y (2021). Analysis of risk factors for perioperative hidden blood loss in unilateral biportal endoscopic spine surgery: a retrospective multicenter study. J Orthop Surg Res.

[27] Ge M, Zhang Y, Ying H, Feng C, Li Y, Tian J, Zhao T, Shao H, Huang Y (2022). Comparison of hidden blood loss and clinical efficacy of percutaneous endoscopic transforaminal lumbar interbody fusion and minimally invasive transforaminal lumbar interbody fusion. Int Orthop.

[28] Yang H, Yang L, Chen D, Wang X, Lu X, Yuan W (2015). Implications of different patterns of "double-layer sign" in cervical ossification of the posterior longitudinal ligament. Eur Spine J.

[29] Li Y, Li J, Wang F, Wang L, Shen Y (2021). Influence of K-line on intraoperative and hidden blood loss in patients with ossification of the posterior longitudinal ligament when undergoing unilateral open-door laminoplasty. J Orthop Surg Res.

[30] Wen L, Jin D, Xie W, Li Y, Chen W, Ding J, Xu J, Ren D (2018). Hidden Blood Loss in Posterior Lumbar Fusion Surgery: An Analysis of Risk Factors. Clin Spine Surg.

[31] Xu S,  Liang Y, Wang J, Yu G, Guo C, Zhu Z, Liu H (2019). Blood Loss of Posterior Lumbar Interbody Fusion on Lumbar Stenosis in Patients With Rheumatoid Arthritis: A Case-Control Study. Spine (Phila Pa 1976).

